# A C57BL/6N mice model of CP/CPPS established by prostate antigen immunization with DPT and BCG co-administration

**DOI:** 10.3389/fimmu.2026.1779951

**Published:** 2026-06-03

**Authors:** Yu Guan, Andong Cheng, Yiding Chen, Hao Li, Feixiang Yang, Wenbo Hao, Qiangsheng Wang, Chaozhao Liang, Jialin Meng

**Affiliations:** 1 Department of Urology, The First Affiliated Hospital of Anhui Medical University; Institute of Urology & Anhui Province Key Laboratory of Urological and Andrological Diseases Research and Medical Transformation, Anhui Medical University, Hefei, Anhui, China; 2 School of Life Sciences, Anhui Medical University, Hefei, China

**Keywords:** BCG, C57BL/6N mice, CP/CPPS, DPT, mice model

## Abstract

**Background and objective:**

Chronic prostatitis/chronic pelvic pain syndrome (CP/CPPS) is common in young and middle-aged men, but existing animal models have key limitations. We aimed to establish a C57BL/6N mice model for mechanistic studies of CP/CPPS.

**Methods:**

We immunized C57BL/6N mice with prostate antigen plus complete Freund’s adjuvant and added DPT, BCG, or DPT+BCG according to group assignment. Von Frey testing, ELISA, flow cytometry, HE staining, and immunohistochemistry were performed to assess pain, cytokines, immune cells, and prostate pathology.

**Key findings and limitations:**

PAg+CFA+DPT+BCG booster immunization induced the strongest pain-related phenotype, increased IL-1β and TNF-α levels, reduced IL-10, aggravated prostatic inflammation, and increased NLRP3, COX-2, NF-κB, and Caspase-1 expression compared with the NC group. Compared with either monotherapy, the PAg+CFA+DPT+BCG group showed stronger inflammatory and pain-related phenotypes across most readouts. Limitation: The model does not fully replicate human CP/CPPS heterogeneity.

**Conclusions and clinical implications:**

We established a C57BL/6N mice model with CP/CPPS-like inflammatory and pain-related features and found that DPT+BCG co-administration produced the strongest phenotype. Its compatibility with transgenic mice may facilitate mechanistic studies and preclinical hypothesis testing in CP/CPPS.

**Patient summary:**

In this study, we established a prostatitis-like model in C57BL/6N mice. Booster immunization with DPT+BCG induced inflammatory and pain-related changes, providing a useful model for future mechanistic studies.

## Introduction

Chronic prostatitis/chronic pelvic pain syndrome (CP/CPPS) is a complex urological disorder characterized by a multifaceted symptom profile, including urogenital pain, lower urinary tract symptoms, psychological comorbidities and sexual dysfunction (1). The impact of CP/CPPS on health-related quality of life (HRQOL) is comparable to that of severe medical conditions such as angina, myocardial infarction, congestive heart failure, diabetes mellitus and Crohn’s disease (2). Although the etiology of prostatitis remains incompletely understood, emerging evidence supports a multifactorial pathogenesis involving intricate interactions between extrinsic and intrinsic factors. These factors include infection, immune dysregulation, lifestyle and dietary influences, psychoneuroendocrine factors and urological abnormalities (3, 4).

Prostatitis animal models serve as indispensable tools for unraveling the cellular and molecular mechanisms that drive disease pathogenesis. They not only facilitate the discovery of novel therapeutic targets but also support the validation of potential intervention strategies. Literature-based testing methods include bacterial and non-bacterial prostatitis animal models (5–7). Researchers have also developed alternative modeling approaches for prostatitis. For example, some investigators established a chronic prostatitis model using the spermine-binding protein (p25) peptide (8). Chuang’s research group demonstrated that intraprostatic capsaicin injection induces neurogenic prostatitis and prostatic pain, suggesting its potential as a valuable research model (9).

The diphtheria, acellular pertussis and tetanus (DPT) vaccine induces both type 1 and type 2 cytokine responses. Both acellular pertussis vaccines and whole-cell pertussis vaccines are capable of inducing T helper cell 17 (Th17) responses (10, 11). Among children who received primary immunization with acellular vaccines, T-cell responses remained at a high level and did not increase following vaccine booster administration. By contrast, in children who received primary immunization with whole-cell vaccines, these responses were amenable to enhancement through vaccine booster administration and natural exposure (12). Subcutaneous inoculation of Bacillus Calmette-Guérin (BCG) induces memory alveolar macrophages (AMs) and trained lung immunity (13).

CFA is a strong adjuvant that may introduce substantial non-specific immune activation, which can complicate mechanistic interpretation (14). In addition, CFA-driven immune responses are predominantly biased toward Th1 and Th17 pathways, resulting in an amplified and non-physiological immune profile (15). The inclusion of DPT further enhances immune activation through heterologous antigen exposure, which may increase background immune complexity and complicate the attribution of observed effects to prostate antigen-specific responses. While such strong stimulation facilitates disease induction, it also reduces experimental resolution when dissecting specific immune mechanisms. Moreover, commonly used models in NOD mice are less convenient for studies requiring extensive genetic manipulation. This significantly limits the feasibility of introducing additional genetic modifications, such as targeted gene knockout or overexpression, thereby restricting the model’s applicability for mechanistic studies. As a result, although the conventional model is effective for inducing prostatitis, its scalability and utility for in-depth molecular investigation remain constrained.

The C57BL/6N background is widely used for generating transgenic and gene-targeted mice lines. Prostate antigen/prostate extract plus CFA is widely recognized as the core framework for EAP induction. Based on previous rodent studies using additional immune-enhancing stimuli, we employed a prostate antigen (PAg)+CFA+DPT protocol as a positive induction reference to evaluate whether the modified strategy could produce comparable EAP-like changes (16–20). In the present study, we tested DPT, BCG, and their combined administration as immune-enhancing interventions in a mice model of chronic prostatitis. By integrating these two vaccines into conventional modeling protocols, we aimed to potentiate immune response activation, ultimately generating a model that recapitulates selected inflammatory and pain-related features of CP/CPPS. Collectively, these findings provide a practical basis for future mechanistic studies of chronic prostatitis in a genetically tractable background.

## Methods

### Animals

Five-week-old male C57BL/6N mice (19 ± 2 g) were purchased from Nanjing Model Animal Center (China) and maintained in the specific pathogen-free animal facility of Anhui Medical University. All procedures involving animals were performed in accordance with the National Institutes of Health Guide for the Care and Use of Laboratory Animals and were approved by the Animal Welfare and Ethics Committee of Anhui Medical University (Approval No. LLSC20232158).

### EAP model induction

We randomly assigned mice to four groups: NC, PAg+CFA+DPT (DPT group), PAg+CFA+BCG (BCG group), and PAg+CFA+DPT+BCG (DPT+BCG group) (n = 5 per group). After arrival, all mice were housed under specific pathogen-free conditions and allowed to acclimatize for 1 week. Mice in the experimental groups then underwent subcutaneous immunization at three sites: the base of the tail (0.05 mL), both footpads (0.025 mL per side), and both shoulder regions (0.025 mL per side). The immunizing emulsion consisted of male rat prostate extract mixed with complete Freund’s adjuvant (CFA; Sigma–Aldrich). Two weeks after the primary immunization, mice were boosted at the same sites with an equal volume of prostate antigen plus CFA. NC group mice received 0.9% saline instead of prostate antigen during both immunizations.

**Figure 1:**
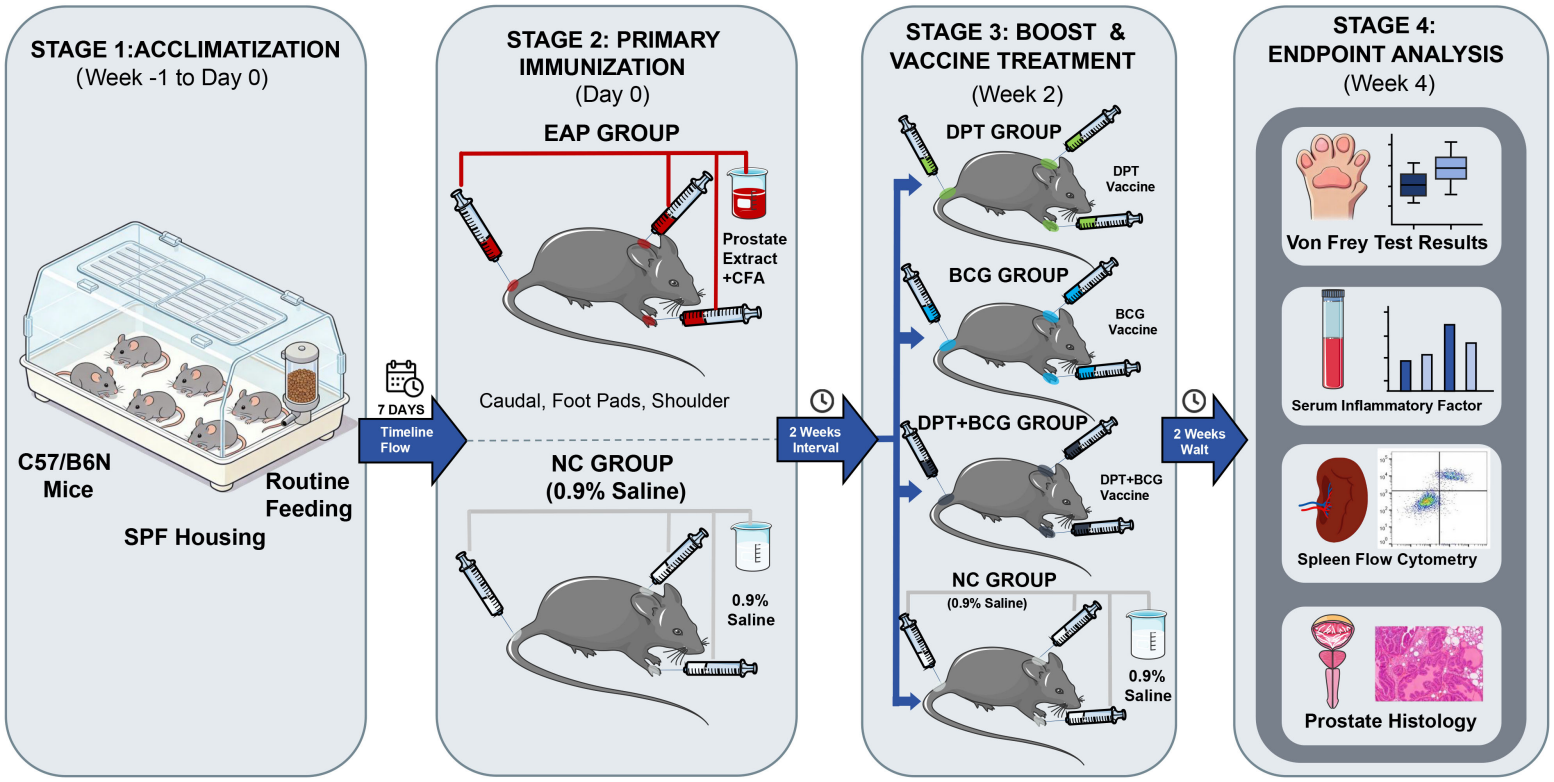
Schematic overview of the experimental design for establishment of the CP/CPPS mice model and endpoint analyses. C57BL/6N mice were housed under specific pathogen-free conditions and allowed to acclimatize for 7 days (Week −1 to Day 0). On Day 0, mice in the experimental groups received primary immunization with prostate extract plus CFA by subcutaneous injection at the caudal region, footpads, and shoulder regions, whereas NC group mice received 0.9% saline. Two weeks later, mice received booster immunization and the corresponding vaccine intervention according to group assignment, including DPT, BCG, or DPT+BCG, while NC group mice received saline. Endpoint analyses were performed at Week 4 and included von Frey behavioral testing, serum inflammatory cytokine measurement, spleen flow cytometry, and prostate histological assessment. Group definitions: NC, saline control; DPT group, PAg+CFA+DPT; BCG group, PAg+CFA+BCG; DPT+BCG group, PAg+CFA+DPT+BCG.

According to group assignment, mice in the DPT, BCG, and DPT+BCG groups additionally received the corresponding vaccine intervention. The DPT vaccine (acellular diphtheria, pertussis, and tetanus vaccine) was obtained from Wuhan Institute of Biological Products Co., Ltd., China (National Medicine Approval No. S19980016). Each dose contained not less than 4.0 IU of acellular pertussis antigen, 30 IU of diphtheria antigen, and 40 IU of tetanus antigen, with aluminum hydroxide as the adjuvant. The vaccine was stored and transported at 2–8 °C, protected from light, and administered at 0.1 mL per mice. The BCG vaccine (live attenuated Mycobacterium bovis BCG vaccine) was purchased from Chengdu Institute of Biological Products Co., Ltd., China (National Medicine Approval No. 20013057). The viable bacterial count was not less than 1.0 × 10^6^ CFU/mg. Excipients included sucrose, gelatin, potassium chloride, and monosodium glutamate. The vaccine was stored at 4 °C for short-term preservation, reconstituted with sterile water for injection immediately before use, and administered at 0.1 mL per mice (**Figure 1**).

### Von frey test

We assessed pelvic pain-related mechanical sensitivity using calibrated von Frey filaments (North Coast Medical, Encinitas, CA, USA). Mice were placed individually in observation chambers and acclimated to the testing environment for 30 min before assessment. The perineal area was selected as the stimulation site. Behavioral testing was performed every 7 days up to Day 28, and the final assessment was conducted immediately before euthanasia. For each testing session, 10 consecutive stimuli were applied bilaterally to the perineum in each animal, and the corresponding withdrawal responses were recorded to compute the response frequency. A positive pain response was defined as the presence of one or more of the following behaviors: vigorous abdominal contraction, immediate licking or grasping of the stimulated site, or jumping (21). All animals were randomly allocated to experimental groups, and behavioral testing was performed by investigators blinded to group assignment.

### Hematoxylin-eosin staining and immunohistochemistry

Following euthanasia, mice prostates were fixed in 10% formalin, then embedded in paraffin and sectioned. HE staining was used to detect prostate inflammation, while the severity was assessed via histological scoring. All evaluations were performed in a double-blind fashion. Specifically, histological scoring was performed independently for five high-power fields, with results presented as the mean value rounded to the nearest integer. Detailed scoring criteria were as follows: Inflammation severity was graded on a 0–3 scale, where a score of 0 indicated no inflammation; 1 denoted mild but definite perivascular mononuclear cell cuffing; 2 represented moderate perivascular mononuclear cell cuffing; and 3 indicated marked perivascular cuffing accompanied by hemorrhage and extensive mononuclear cell infiltration in the parenchyma (22).

Immunohistochemical analyses were performed on paraffin-embedded sections. Sections were first dewaxed in xylene, followed by hydration in graded ethanol solutions. Antigen retrieval was performed using EDTA-containing antigen repair solution, and endogenous peroxidase activity was then quenched. We used primary antibodies against the following proteins: Foxp3 (AF6544, 1:100, Affinity, USA), CD68 (AB303565, 1:1000, Abcam, USA), NLRP3 (PA5-79740, 1:400, Thermo Fisher Scientific, USA), COX-2 (ABB8852, 1:200, Huilanbio, China), NF-κB (ABB5034, 1:200, Huilanbio, China), and Caspase-1 (AF5418, 1:100, Affinity, USA). Sections were incubated overnight at 4 °C with the corresponding primary antibodies. Subsequently, appropriate secondary antibodies were applied and incubated for 2 hours at room temperature in the dark. Finally, sections were imaged using a digital slide scanner (Pannoramic MIDI, 3DHistech, Hungary).

### Flow cytometry analysis

We quantified the proportion of Th17 cells by flow cytometry. After euthanasia, we harvested spleens from the mice and homogenized them in PBS. The resulting cell suspension was treated with red blood cell lysate and washed twice with PBS. Cells were then transferred to flow cytometry tubes, and FITC-CD4 surface antibody (rat anti-mice; BD, 553046, USA) was added for incubation. Following another PBS wash, cells were stimulated by incubation at 37 °C in RPMI 1640 medium (Gibco) containing phorbol 12-myristate 13-acetate (PMA), ionomycin and monensin (MultiSciences, China). Subsequently, cells were subjected to membrane permeabilization and fixation. Intracellular PE-IL17A antibody (rat anti-mice; BD, 559502) was then added, followed by incubation at 4 °C for 1 hour. After final washes and cell resuspension, samples were analyzed using a FACSCalibur flow cytometer (Beckman Coulter), and the resulting data were processed with analytical software.

### Enzyme-linked immunosorbent assay

Enzyme-linked immunosorbent assay (ELISA) was used to quantify cytokine levels, including IL-1β, TNF-α and IL-10 in serum. All assays were performed according to the manufacturers’ protocols using the respective kits: IL-1β (mlbio, ml098416M, China), TNF-α (mlbio, ml002095-2, China), and IL-10 (mlbio, ml037873-2, China).

### Statistics

Data are presented as Mean ± SD. Statistical analyses were performed using SPSS version 21.0 (IBM Corp., Armonk, NY, USA). Comparisons among multiple groups were conducted using one-way or two-way ANOVA followed by Tukey’s *post hoc* test. Normality of distribution and homogeneity of variance were assessed prior to analysis. Nonparametric tests were used if data were not normally distributed. We considered two-sided P values < 0.05 statistically significant.

## Results

### Histopathology

**Figure 2:**
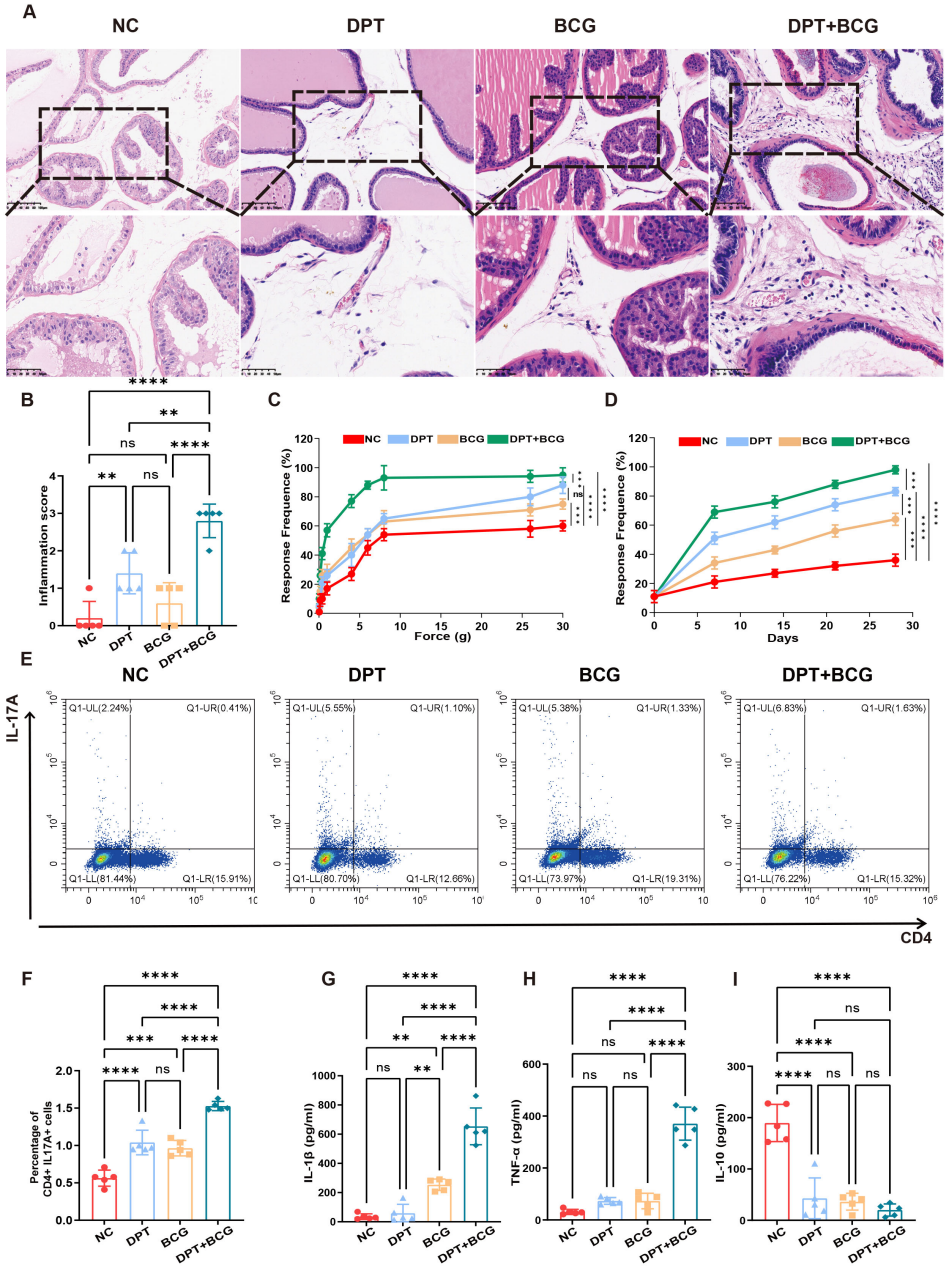
Inflammatory cell infiltration, pain assessment, inflammatory cytokine levels, and CD4^+^IL-17A^+^ Th17 cell proportions across four groups **(A, B)** Inflammatory cell infiltration in prostate tissues of the four groups. Varying degrees of inflammatory cell infiltration were observed in the stroma of prostate tissues across the three model groups, with the most severe infiltration noted in the DPT+BCG group. **(C, D)** Pelvic pain responsiveness in the four groups of mice. The DPT+BCG group exhibited the most pronounced hypersensitivity. **(E, F)** Flow cytometry analysis revealed that the proportion of splenic CD4^+^IL-17A^+^ Th17 cells was significantly higher in all three model groups compared to the control group. No statistically significant difference in cell proportions was observed between the BCG and DPT monotherapy groups, whereas the DPT+BCG group exhibited the highest proportion of these cells. **(G-I)** Concentrations of IL-1β, TNF-α, and IL-10 in peripheral blood of the four groups. Levels of IL-1β and TNF-α in the three experimental groups were significantly higher than those in the control group, whereas IL-10 levels showed the opposite trend. Data are presented as Mean ± SD. **p < 0.01, ***p < 0.001, ****p < 0.0001; ns, not significant. Group definitions: NC, saline control; DPT group, PAg+CFA+DPT; BCG group, PAg+CFA+BCG; DPT+BCG group, PAg+CFA+DPT+BCG.

Histological examination showed inflammatory cell infiltration and neovascularization in all three immunized groups compared with the NC group. These changes were most pronounced in the DPT+BCG group, which showed dense inflammatory infiltration and more obvious structural disruption of the prostate tissue (**Figures 2A, B**). One-way ANOVA showed a significant treatment effect on inflammation score, *F*(3,6) = 26.33, *p* < 0.001, *R^2^* = 0.83. The inflammation score was highest in the DPT+BCG group (2.80 ± 0.45), followed by the DPT group (1.40 ± 0.55), the BCG group (0.60 ± 0.55), and the NC group (0.20 ± 0.45). Tukey’s *post hoc* analysis showed that the DPT+BCG group had significantly higher scores than the NC group (*p* < 0.0001), the BCG group (*p* < 0.0001) and the DPT group (*p* = 0.0022). The BCG group did not differ significantly from the NC or DPT groups (both *p* > 0.05).

### Von frey behavioral assessment

Response frequency differed markedly among groups (**Figures 2C, D**). The response frequency increased with stimulus force in all groups. The DPT+BCG group showed significantly higher response frequency than the NC, DPT, and BCG groups at all force values (*p* < 0.0001), whereas the DPT and BCG groups did not differ significantly from each other (*p* = 0.8665). The NC group showed the lowest response frequency across all force levels. Response frequency also increased over time in all groups, and the DPT+BCG group remained the most responsive throughout the observation period. Two-way ANOVA confirmed significant effects of force or time, treatment, and their interaction on response frequency (all *p* < 0.0001).

### Proportion of Th17 cells across groups

Flow cytometry showed significant differences in the proportion of splenic CD4^+^IL-17A^+^ Th17 cells among the four groups. Representative dot plots and quantitative analysis are shown in **Figures 2E, F**. One-way ANOVA confirmed a highly significant difference in the percentage of Th17 cells across groups, *F*(3, 16) = 59.41, *p* < 0.0001, *R^2^* = 0.92. The DPT+BCG group exhibited the highest Th17 cell proportion (1.53 ± 0.06%), which was significantly greater than that in the NC group (0.56 ± 0.11%, *p* < 0.0001), DPT group (1.04 ± 0.16%, *p* < 0.0001), and BCG group (0.96 ± 0.10%, *p* < 0.0001). Both the DPT and BCG groups showed significantly higher Th17 cell percentages than the NC group (*p* < 0.0001 and *p* = 0.0003, respectively), while no statistically significant difference was detected between the DPT and BCG groups (*p* = 0.7251).

### Serum levels of TNF-α, IL-1β, IL-10

Serum IL-1β levels differed significantly among groups in **Figure 2G**. An ordinary one-way ANOVA revealed a significant treatment effect (*F*(3, 16) = 76.17, *p* < 0.0001, *R^2^* = 0.93). *Post hoc* Tukey’s multiple comparisons test demonstrated the following: The DPT+BCG group exhibited the highest IL-1β concentration (653.70 ± 125.80 pg/mL), which was significantly greater than that in the NC group (35.00 ± 19.62 pg/mL, *p* < 0.0001), DPT group (58.58 ± 61.35 pg/mL, *p* < 0.0001), and BCG group (253.70 ± 39.21 pg/mL, *p* < 0.0001). The BCG group showed significantly higher IL-1β levels than both the NC group (*p* = 0.0012) and the DPT group (*p* = 0.0034). No statistically significant difference was observed between the NC and DPT groups (*p* = 0.9559). Similarly, group differences in serum TNF-α levels were significant (*F*(3, 16) = 94.69, *p* < 0.0001, *R^2^* = 0.95) in **Figure 2H**. Tukey’s post−hoc tests showed that TNF−α in the DPT+BCG group (370.80 ± 63.74 pg/mL) was significantly higher than in the NC (31.06 ± 9.57 pg/mL, *p* < 0.0001), DPT (73.37 ± 13.02 pg/mL, *p* < 0.0001), and BCG (73.23 ± 29.73 pg/mL, *p* < 0.0001) groups. No significant differences were detected among the NC, DPT and BCG groups. Conversely, IL-10 levels were highest in the NC group with a significant group difference, *F*(3, 16) = 37.69, *p* < 0.0001, *R^2^* = 0.88. The NC group had the highest level (189.7 ± 36.23 pg/mL), which was significantly greater than the DPT (42.84 ± 39.80 pg/mL), BCG (36.17 ± 16.42 pg/mL), and DPT+BCG (20.04 ± 11.82 pg/mL) groups (all *p* < 0.0001). No significant differences were detected among the three treatment groups (**Figure 2I**).

### Expression profiles of specific proteins in macrophages and regulatory T cells

**Figure 3:**
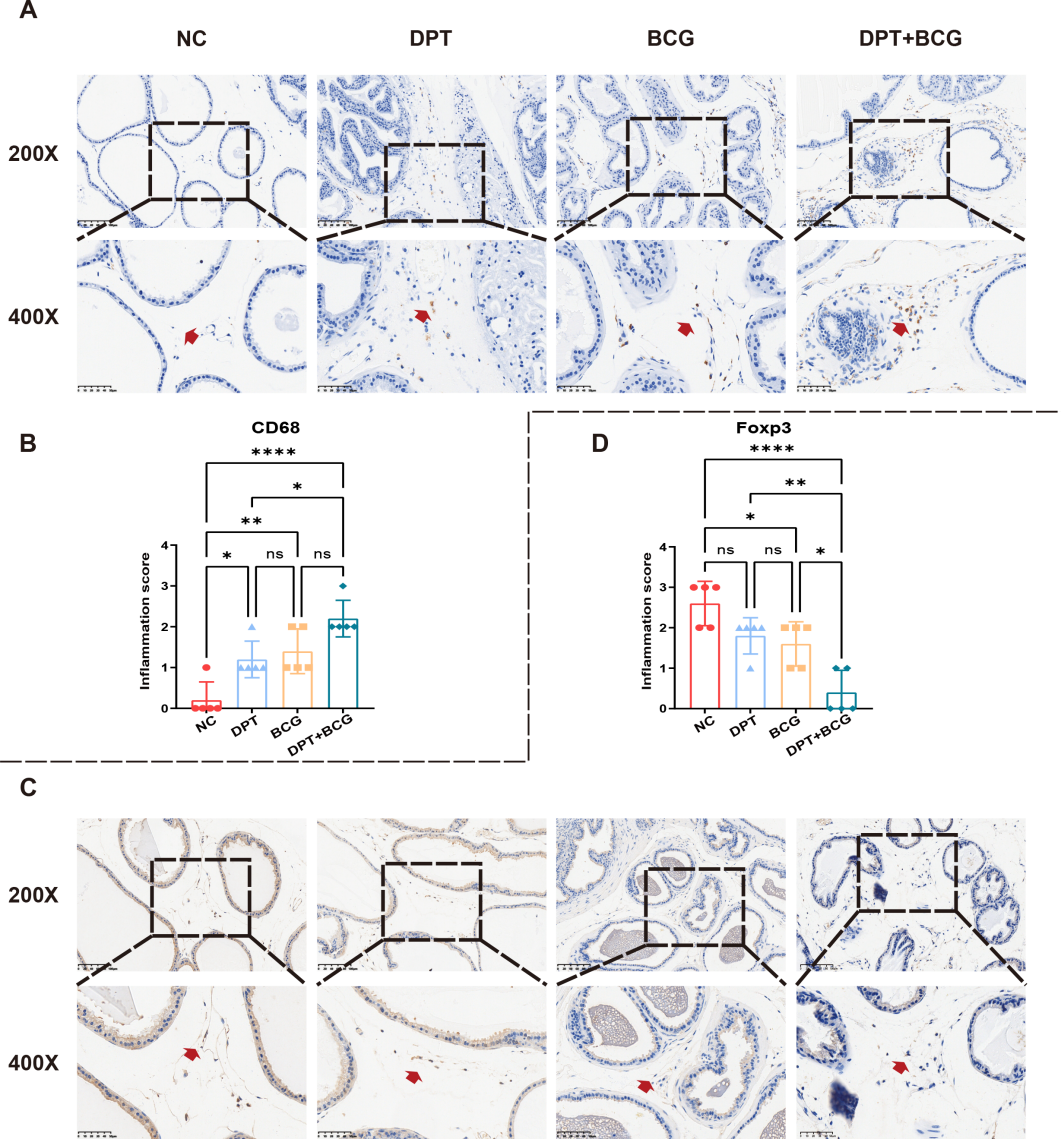
Expression of CD68 (macrophage marker) and Foxp3 (Treg marker) Proteins in prostate tissues among four groups. **(A, B)** CD68 protein expression in the four groups. CD68 expression was significantly higher in the model groups than in the control group, with the highest level observed in the DPT+BCG group. **(C, D)** Foxp^3^ protein expression in the four groups. Foxp^3^ expression was higher in the control group compared to the model groups, and the lowest level was noted in the DPT+BCG group. Data are presented as Mean ± SD. *p < 0.05, **p < 0.01, ****p < 0.0001; ns, not significant. Group definitions: NC, saline control; DPT group, PAg+CFA+DPT; BCG group, PAg+CFA+BCG; DPT+BCG group, PAg+CFA+DPT+BCG.

Immunohistochemical analysis was performed using CD68 and Foxp^3^ as markers for macrophages and Treg cells, respectively. CD68 staining increased in the treatment groups and was strongest in the DPT+BCG group. The DPT and BCG monotherapy groups also showed increased staining, although less prominently than the co-immunization group. Consistent with the histological localization, the staining score based on CD68 expression was significantly elevated in the treatment groups (*F*(3, 16) = 15.04, *p* < 0.0001, *R^2^* = 0.74). Specifically, the DPT+BCG group displayed the highest score (2.20 ± 0.45), which was significantly higher than the NC (0.20 ± 0.45, *p* < 0.0001), DPT (1.20 ± 0.45, *p* = 0.0051), and BCG (1.40 ± 0.55, *p* = 0.0198) groups. No significant difference was observed between the DPT and BCG groups (**Figures 3A, B**). In contrast, Foxp3 staining was more pronounced in the interstitial spaces of prostate tissues from the NC group. Foxp3 scores showed an opposing trend, with a significant group difference, *F*(3, 16) = 15.03, *p* < 0.0001, *R²* = 0.74. The NC group showed the highest staining score (2.60 ± 0.55), which was significantly higher than the BCG and DPT+BCG groups (all *p* < 0.05). The DPT+BCG group had the lowest score, which was significantly lower than the DPT and BCG groups (all *p* < 0.05), with no differences between the NC and DPT, or DPT and BCG groups (**Figures 3C, D**).

### Expression profiles of inflammation- and apoptosis-associated proteins

**Figure 4:**
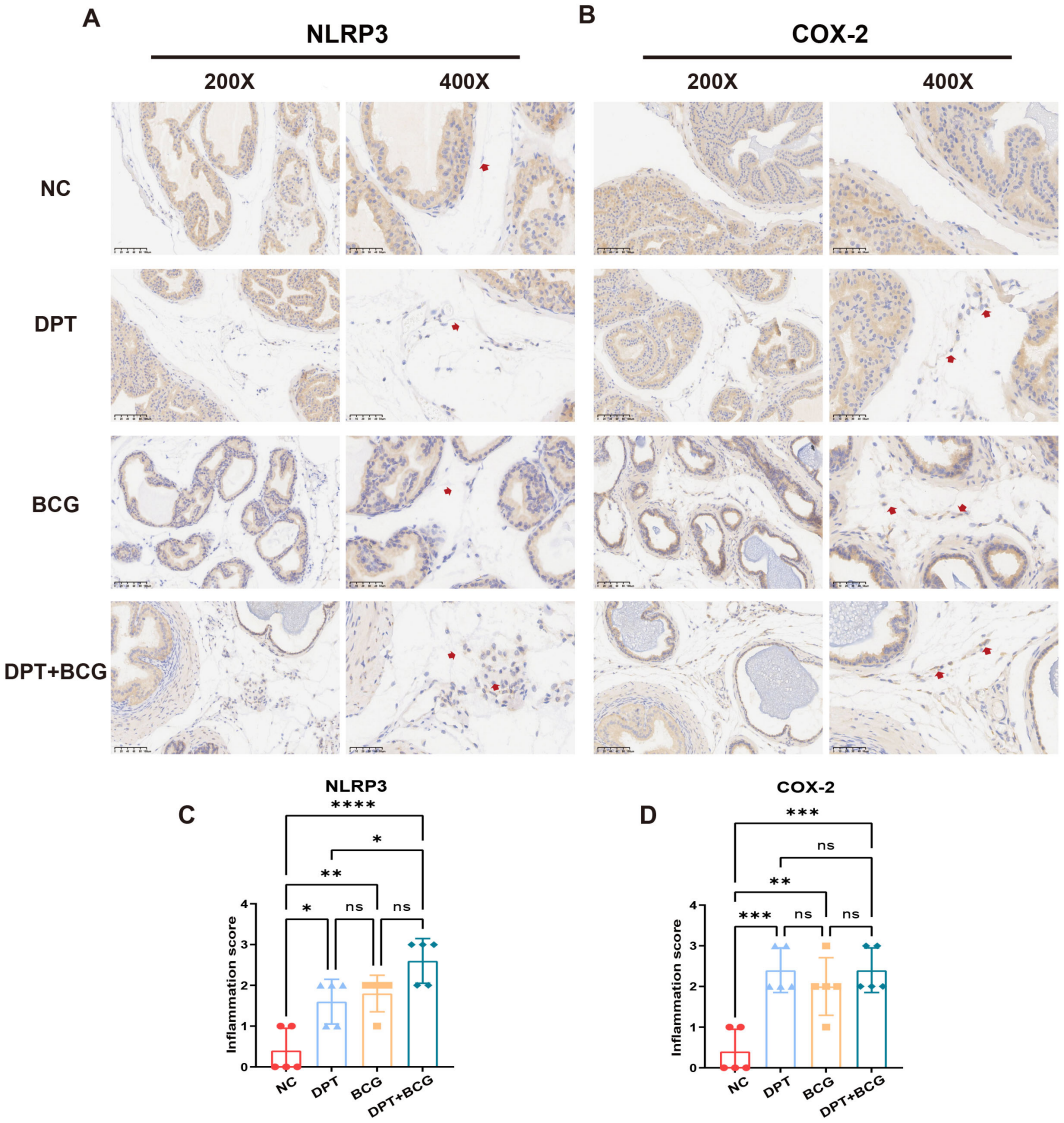
Expression of NLRP3 and COX-2 in prostate tissues from the four experimental groups. **(A, B)** Representative immunohistochemical staining images of NLRP3 **(A)** and COX-2 **(B)** in prostate tissues from the NC, DPT, BCG, and DPT+BCG groups at 200x and 400x magnification. Positive staining is indicated by red arrows. **(C, D)** Semiquantitative analysis of NLRP3 **(C)** and COX-2 **(D)** staining scores in the four groups. NLRP3 and COX-2 expression increased in the treatment groups compared with the NC group, with the highest levels observed in the DPT+BCG group. Data are presented as Mean ± SD. *p < 0.05, **p < 0.01, ***p < 0.001, ****p < 0.0001; ns, not significant. Group definitions: NC, saline control; DPT group, PAg+CFA+DPT; BCG group, PAg+CFA+BCG; DPT+BCG group, PAg+CFA+DPT+BCG.

**Figure 5:**
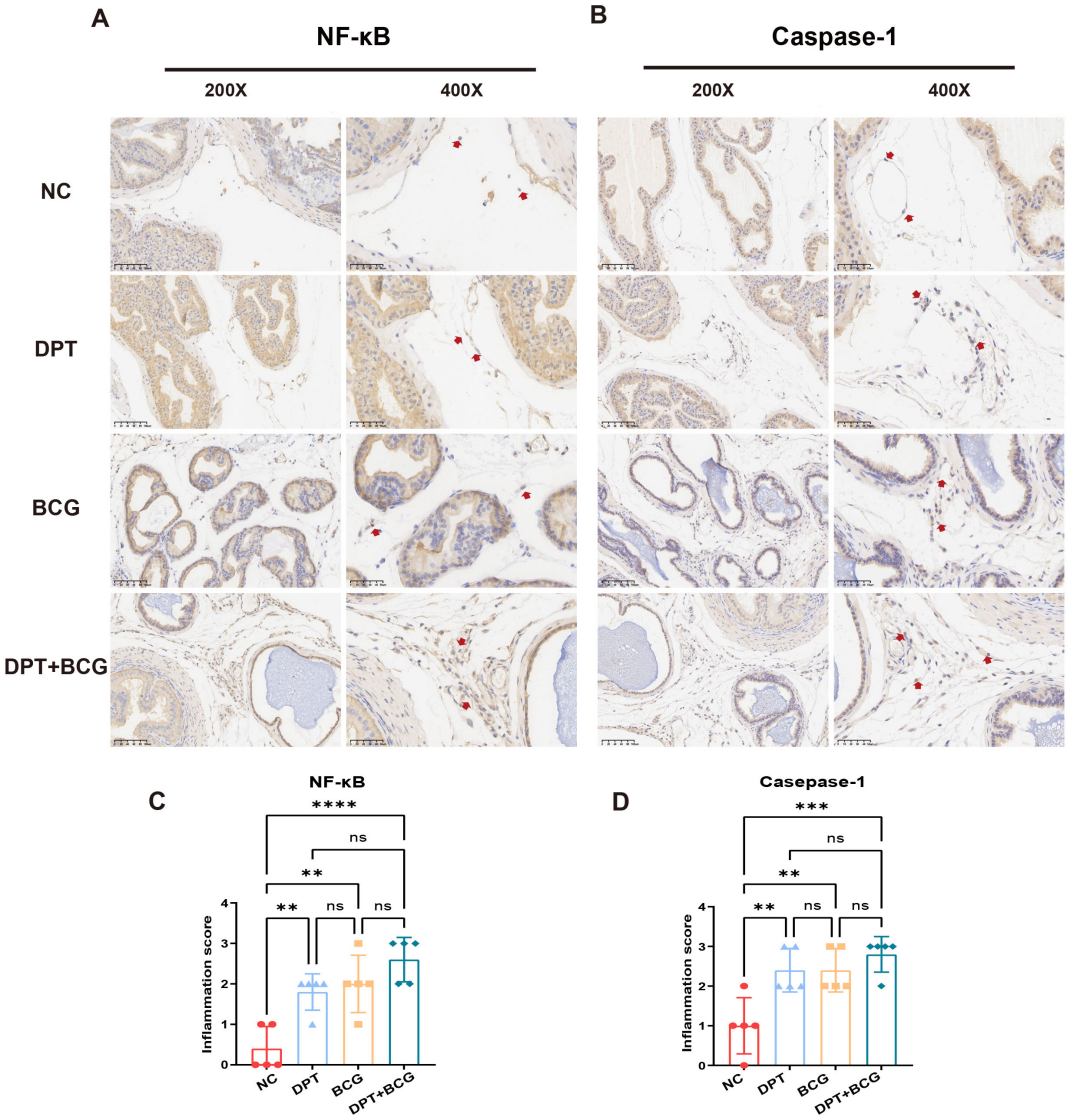
Expression of NF-κB and Caspase-1 in prostate tissues from the four experimental groups. **(A, B)** Representative immunohistochemical staining images of NF-κB **(A)** and Caspase-1 **(B)** in prostate tissues from the NC, DPT, BCG, and DPT+BCG groups at 200× and 400× magnification. Positive staining is indicated by red arrows. **(C, D)** Semiquantitative analysis of NF-κB **(C)** and Caspase-1 **(D)** staining scores in the four groups. NF-κB and Caspase-1 expression was higher in the treatment groups than in the NC group, and the DPT+BCG group showed the strongest staining intensity overall. Data are presented as mean ± SD. **: p<0.01, ****: p<0.0001, ns: no significance. Group definitions: NC, saline control; DPT group, PAg+CFA+DPT; BCG group, PAg+CFA+BCG; DPT+BCG group, PAg+CFA+DPT+BCG.

Because evaluation of EAP models generally depends on integrated evidence, including histopathological changes and inflammation-associated readouts, the present study interpreted NLRP3, COX-2, NF-κB, and Caspase-1 primarily as auxiliary markers reflecting prostate inflammatory responses after model induction. NLRP3 expression was significantly upregulated in treatment groups, *F*(3, 16) = 15.03, *p* < 0.0001, *R^2^* = 0.74. Consistent with the pro-inflammatory phenotype, NLRP3 expression was highest in the DPT+BCG group (2.60 ± 0.55), which was significantly higher than the NC (0.40 ± 0.55, *p* < 0.0001) and DPT (1.60 ± 0.55, *p* < 0.05) groups. Both the DPT and BCG (1.80 ± 0.45) groups showed significantly elevated scores compared to the NC group (*p* < 0.05 and *p* < 0.01, respectively), with no significant differences between the DPT and BCG, or BCG and DPT+BCG groups (**Figures 4A, C**). COX-2 levels differed significantly across groups, *F*(3, 16) = 12.95, *p* = 0.0002, *R²* = 0.71. DPT and DPT + BCG groups had the highest scores (2.40 ± 0.55 for both), significantly higher than NC (0.40 ± 0.55, *p* = 0.0003). BCG (2.00 ± 0.71) also showed a higher score than NC (*p* = 0.0029), with no differences among DPT, BCG, and DPT+BCG (**Figures 4B, D**). NF-κB expression was significantly elevated in treatment groups, *F*(3, 16) = 13.33, *p* = 0.0001, *R²* = 0.71. The DPT+BCG group had the highest score (2.60 ± 0.55), significantly higher than NC (0.40 ± 0.55, *p* < 0.0001), DPT (1.80 ± 0.45, *p* = 0.0065), and BCG (2.00 ± 0.71, *p* = 0.0021). Both DPT and BCG groups showed higher scores than NC, with no differences among treatment groups (**Figures 5A, C**). For Caspase−1, group differences were significant (F(3, 16) = 9.590, p = 0.0007). The DPT+BCG group had the highest staining score (2.80 ± 0.45), significantly higher than the NC group (1.00 ± 0.71, p < 0.01). Both the DPT and BCG groups showed higher scores than the NC group (p < 0.01), with no differences among treatment groups (**Figures 5B, D**).

## Discussion

CP/CPPS is highly prevalent in clinical settings, yet it is frequently misdiagnosed and suboptimally managed. This often results in persistent clinical manifestations, considerable morbidity, and substantial patient dissatisfaction (23). A dysregulated inflammatory response driven by autoimmune mechanisms has been proposed as a key contributor to disease initiation and progression (24). The autoimmune hypothesis carries considerable significance for elucidating the pathogenesis of CP/CPPS, with robust support from clinical studies conducted in patients and preclinical investigations utilizing animal models. Furthermore, it has been proposed that prostatitis can lead to the persistence of chronic pelvic pain and impair semen quality, thereby affecting male fertility (18, 25, 26). In our study, pain responses were notably enhanced in all three immunized model groups relative to the NC group. Of particular note, the DPT+BCG co-immunization group exhibited the most prominent pain-related behaviors.

The sustained activation of the inflammatory cascade drives the continuous production of pro-inflammatory mediators, including cytokines, chemokines and reactive oxygen species (ROS). Accumulating evidence from clinical studies has demonstrated that seminal plasma samples obtained from patients with type IIIa prostatitis display statistically significant elevations in the concentrations of IL-1β, TNF-α, IL-6 and IL-8 relative to samples from healthy control individuals. In contrast, semen from type IIIb prostatitis patients showed increased IL-1β, TNF-α, and IL-8 levels, whereas IL-6 levels remained unchanged (27). Our results showed no statistically significant difference in IL-1β levels between the DPT group and the NC group, whereas the DPT+BCG group had the highest IL-1β levels. Similarly, TNF-α levels peaked in the DPT+BCG group, whereas the DPT and BCG groups showed no clear separation from the NC group, with little variation between the DPT and BCG groups. In contrast, the concentrations of IL-10 were notably reduced in all experimental groups relative to the NC group and the DPT+BCG combination group exhibited the lowest levels among all groups. This indicates a more pronounced inflammatory response in the co-immunization group than in the single-immunization groups.

The sustained autoimmune inflammation is thought to stem from the persistent expression of self-antigens coupled with insufficient quantities of regulatory immune cell subsets. This immune dysregulation often skews the immune response toward a destructive phenotype dominated by Th1 and Th17 cells. Some secretions further amplify inflammatory cascades and activate additional immune cells, particularly T lymphocytes. Accumulating evidence has firmly established Th1 and Th17 cells as pivotal mediators in the pathogenesis of numerous autoimmune disorders (28, 29). Our results demonstrated a significant elevation in the proportion of splenic Th17 cells across all experimental groups relative to the NC group while the DPT+BCG co-immunization group exhibited the highest Th17 cell ratio.

Histological examinations showed that the DPT+BCG co-immunization group had denser inflammatory infiltration and higher expression of inflammatory-related proteins than the DPT and BCG monotherapy groups. These findings are consistent with the cytokine and immune cell data and support a stronger pro-inflammatory phenotype after co-immunization. Our model uses the C57BL/6N background and incorporates BCG into the antigen+CFA+DPT framework, thereby enhancing both experimental flexibility and mechanistic applicability. BCG has been shown to induce trained immunity through epigenetic and functional reprogramming of innate immune cells, providing a sustained and enhanced inflammatory response that complements the adaptive immune activation induced by DPT and antigen-specific stimulation [15. Rather than reducing immune complexity, this model integrates multiple layers of immune activation into a structured and experimentally tractable system. Importantly, the use of C57BL/6N mice provides a major technical advantage. As the most widely used background for genetically engineered mice models, C57BL/6N enables seamless integration with extensive transgenic and knockout mice resources. This allows for efficient implementation of additional genetic modifications, facilitating systematic dissection of immune pathways involved in chronic prostatitis.

This study has several limitations. We did not capture the full clinical heterogeneity of chronic prostatitis, and our analysis focused on inflammatory phenotypes and a limited set of inflammatory proteins rather than broader molecular pathways. We also did not directly compare this model with conventional NOD-based EAP models, and therefore cannot claim superiority over existing systems. In addition, mice were evaluated 2 weeks after the second immunization, which reflects an early chronic or subacute stage rather than a definitively established long-term chronic phase. Thus, the present data do not yet demonstrate sustained phenotypic persistence. Serum cytokine changes and pain-related behavior also cannot fully distinguish organ-specific autoimmune prostatitis from non-specific pain sensitization associated with systemic inflammation. Although prostate histology and local inflammatory protein expression support prostatitis-like tissue involvement, they do not completely exclude a contribution from vaccine-driven systemic inflammatory sensitization. Finally, the sample size was relatively small. Further studies with larger cohorts, later time points, and broader mechanistic analyses will be needed to refine and validate this model. Despite these limitations, the model offers a useful platform for mechanistic studies in a genetically tractable background.

## Conclusions

We established a C57BL/6N mice model with CP/CPPS-like inflammatory and pain-related features and found that DPT+BCG co-administration produced the strongest phenotype. This model may support mechanistic studies and preclinical hypothesis testing in CP/CPPS, particularly in genetically engineered mice.

## Data Availability

The original contributions presented in the study are included in the article/supplementary material. Further inquiries can be directed to the corresponding authors.

## References

[1] Sun Y Liu Y Liu B Zhou K Yue Z Zhang W . Efficacy of acupuncture for chronic prostatitis/chronic pelvic pain syndrome: a randomized trial. Ann Intern Med. (2021) 174:1357–66. doi: 10.1177/09645284251379493. PMID: 34399062

[2] Rees J Abrahams M Doble A Cooper A Prostatitis Expert Reference G . Diagnosis and treatment of chronic bacterial prostatitis and chronic prostatitis/chronic pelvic pain syndrome: a consensus guideline. BJU Int. (2015) 116:509–25. doi: 10.1111/bju.13101. PMID: 25711488 PMC5008168

[3] Wang X Zhong S Xu T Xia L Zhang X Zhu Z . Histopathological classification criteria of rat model of chronic prostatitis/chronic pelvic pain syndrome. Int Urol Nephrol. (2015) 47:307–16. doi: 10.1016/s1569-9056(18)31797-4 25409932

[4] De Marzo AM Nakai Y Nelson WG . Inflammation, atrophy, and prostate carcinogenesis. Urol Oncol. (2007) 25:398–400. doi: 10.1016/j.urolonc.2007.05.007. PMID: 17826659

[5] De Marzo AM Platz EA Sutcliffe S Xu J Gronberg H Drake CG . Inflammation in prostate carcinogenesis. Nat Rev Cancer. (2007) 7:256–69. doi: 10.21236/ada494446 PMC3552388 17384581

[6] Vykhovanets EV Resnick MI MacLennan GT Gupta S . Experimental rodent models of prostatitis: limitations and potential. Prostate Cancer Prostatic Dis. (2007) 10:15–29. doi: 10.1038/sj.pcan.4500930. PMID: 17199136

[7] Li TF Li WW Xia XY Huang YF . Animal models of chronic prostatitis: an update. Zhonghua Nan Ke Xue. (2013) 19:1124–8. 24432628

[8] Altuntas CZ Daneshgari F Veizi E Izgi K Bicer F Ozer A . A novel murine model of chronic prostatitis/chronic pelvic pain syndrome (CP/CPPS) induced by immunization with a spermine binding protein (p25) peptide. Am J Physiol Regul Integr Comp Physiol. (2013) 304:R415–422. doi: 10.1152/ajpregu.00147.2012. PMID: 23344231 PMC3602823

[9] Chuang YC Yoshimura N Wu M Huang CC Chiang PH Tyagi P . Intraprostatic capsaicin injection as a novel model for nonbacterial prostatitis and effects of botulinum toxin A. Eur Urol. (2007) 51:1119–27. doi: 10.1016/j.eururo.2006.11.037. PMID: 17141941

[10] Ausiello CM Urbani F la Sala A Lande R Cassone A . Vaccine- and antigen-dependent type 1 and type 2 cytokine induction after primary vaccination of infants with whole-cell or acellular pertussis vaccines. Infect Immun. (1997) 65:2168–74. doi: 10.1128/iai.65.6.2168-2174.1997. PMID: 9169747 PMC175299

[11] Ross PJ Sutton CE Higgins S Allen AC Walsh K Misiak A . Relative contribution of Th1 and Th17 cells in adaptive immunity to Bordetella pertussis: towards the rational design of an improved acellular pertussis vaccine. PloS Pathog. (2013) 9:e1003264. doi: 10.1371/journal.ppat.1003264. PMID: 23592988 PMC3617212

[12] Edwards KM Berbers GA . Immune responses to pertussis vaccines and disease. J Infect Dis. (2014) 209:S10–15. doi: 10.1093/infdis/jit560. PMID: 24158958

[13] Chen J Gao L Wu X Fan Y Liu M Peng L . BCG-induced trained immunity: history, mechanisms and potential applications. J Transl Med. (2023) 21:106. doi: 10.1186/s12967-023-03944-8. PMID: 36765373 PMC9913021

[14] Lazarević M Stanisavljević S Nikolovski N Dimitrijević M Miljković Đ . Complete Freund’s adjuvant as a confounding factor in multiple sclerosis research. Front Immunol. (2024) 15. doi: 10.3389/fimmu.2024.1353865 PMC10902151 38426111

[15] Mone K Singh S Abdullatif F Sur M Rasquinha MT Seravalli J . Immunization with complete Freund’s adjuvant reveals trained immunity-like features in A/J mice. Vaccines. (2025) 13(7):768. doi: 10.3390/vaccines13070768. PMID: 40733745 PMC12300380

[16] Manuel RSJ . Trends in experimental autoimmune prostatitis: insights into pathogenesis, therapeutic strategies, and redefinition. Am J Clin Exp Urol. (2024) 12:52–63. doi: 10.62347/oujj3710. PMID: 38736617 PMC11087208

[17] He H Luo H Xu H Qian B Zou X Zhang G . Preclinical models and evaluation criteria of prostatitis. Front Immunol. (2023) 14:1183895. doi: 10.3389/fimmu.2023.1183895. PMID: 37228599 PMC10203503

[18] Wang W Naveed M Baig MMFA Abbas M Xiaohui Z . Experimental rodent models of chronic prostatitis and evaluation criteria. Biomed Pharmacother. (2018) 108:1894–901. doi: 10.1016/j.biopha.2018.10.010. PMID: 30453450

[19] He Y Zeng H Yu Y Zhang J Duan X Liu Q . Resveratrol improves smooth muscle carcinogenesis in the progression of chronic prostatitis via the downregulation of c-kit/SCF by activating Sirt1. Biomed Pharmacother. (2017) 95:161–6. doi: 10.1016/j.biopha.2017.08.064. PMID: 28841456

[20] Zhang J Yi QT Gong M Zhang YQ Liu D Zhu RJ . Upregulation of TRPV1 in spinal dorsal root ganglion by activating NGF‐TrkA pathway contributes to pelvic organ cross‐sensitisation in rats with experimental autoimmune prostatitis. Andrologia. (2019) 51(8):e13302. doi: 10.1111/and.13302. PMID: 31074030

[21] Du H Chen X Zhang L Liu Y Zhan C Chen J . Experimental autoimmune prostatitis induces learning-memory impairment and structural neuroplastic changes in mice. Cell Mol Neurobiol. (2020) 40:99–111. doi: 10.1007/s10571-019-00723-2. PMID: 31401743 PMC11448931

[22] Breser ML Motrich RD Sanchez LR Rivero VE . Chronic pelvic pain development and prostate inflammation in strains of mice with different susceptibility to experimental autoimmune prostatitis. Prostate. (2017) 77:94–104. doi: 10.1002/pros.23252. PMID: 27699823

[23] Pendegast HJ Leslie SW Rosario DJ . Chronic prostatitis and chronic pelvic pain syndrome in men. In: StatPearls. Treasure Island (FL): StatPearls Publishing. (2025). 38261706

[24] Salazar FC Martinez MS Paira DA Chocobar YA Olivera C Godoy GJ . CD8 T cells are dispensable for experimental autoimmune prostatitis induction and chronic pelvic pain development. Front Immunol. (2024) 15:1387142. doi: 10.3389/fimmu.2024.1387142. PMID: 38807587 PMC11130463

[25] Breser ML Salazar FC Rivero VE Motrich RD . Immunological mechanisms underlying chronic pelvic pain and prostate inflammation in chronic pelvic pain syndrome. Front Immunol. (2017) 8:898. doi: 10.3389/fimmu.2017.00898. PMID: 28824626 PMC5535188

[26] Verze P Cai T Lorenzetti S . The role of the prostate in male fertility, health and disease. Nat Rev Urol. (2016) 13:379–86. doi: 10.1038/nrurol.2016.89. PMID: 27245504

[27] Orhan I Onur R Ilhan N Ardicoglu A . Seminal plasma cytokine levels in the diagnosis of chronic pelvic pain syndrome. Int J Urol. (2001) 8:495–9. doi: 10.1046/j.1442-2042.2001.00358.x. PMID: 11683970

[28] Sehrawat S Rouse BT . Interplay of regulatory T cell and Th17 cells during infectious diseases in humans and animals. Front Immunol. (2017) 8:341. doi: 10.3389/fimmu.2017.00341. PMID: 28421070 PMC5377923

[29] Sharif K Amital H Shoenfeld Y . The role of dietary sodium in autoimmune diseases: the salty truth. Autoimmun Rev. (2018) 17:1069–73. doi: 10.1016/j.autrev.2018.05.007. PMID: 30213699

